# Deep Learning Methods for Heart Sounds Classification: A Systematic Review

**DOI:** 10.3390/e23060667

**Published:** 2021-05-26

**Authors:** Wei Chen, Qiang Sun, Xiaomin Chen, Gangcai Xie, Huiqun Wu, Chen Xu

**Affiliations:** 1 Medical School, Nantong University, Nantong 226001, China; chenwei0303@ntu.edu.cn (W.C.); gangcai@ntu.edu.cn (G.X.); wuhuiqun@ntu.edu.cn (H.W.); 2 School of Information Science and Technology, Nantong University, Nantong 226019, China; chenxm@ntu.edu.cn

**Keywords:** CVDs, CNN, deep learning, heart sounds classification, RNN

## Abstract

The automated classification of heart sounds plays a significant role in the diagnosis of cardiovascular diseases (CVDs). With the recent introduction of medical big data and artificial intelligence technology, there has been an increased focus on the development of deep learning approaches for heart sound classification. However, despite significant achievements in this field, there are still limitations due to insufficient data, inefficient training, and the unavailability of effective models. With the aim of improving the accuracy of heart sounds classification, an in-depth systematic review and an analysis of existing deep learning methods were performed in the present study, with an emphasis on the convolutional neural network (CNN) and recurrent neural network (RNN) methods developed over the last five years. This paper also discusses the challenges and expected future trends in the application of deep learning to heart sounds classification with the objective of providing an essential reference for further study.

## 1. Introduction

With increasing industrialization, urbanization, and globalization, cardiovascular diseases (CVDs) are posing a serious threat to human health, causing the death of increasing numbers of people globally. Approximately 17.9 million people died from CVDs in 2016, accounting for 31% of all global deaths. Of these deaths, 85% resulted from heart attack and stroke [1]. CVDs exert a heavy burden on the finances of sufferers in low- and middle-income countries, and early detection and diagnosis are very significant to reducing the mortality rate. Cardiac auscultation is a simple, essential, and efficient method for examining CVDs and has a history of more than 180 years [2]. It is crucial to the early diagnosis of CVDs because of its noninvasiveness and good performance for reflecting the mechanical motion of the heart and cardiovascular system. However, cardiac auscultation requires substantial clinical experience and skill, and the human ear is not sensitive to sounds within all frequency ranges. The use of computers for the automatic analysis and classification of heart sound signals promises to afford substantial improvements in this area of human health management.

A heart sound is a kind of physiological signal, and its measurement is known as phonocardiography (PCG). It is produced by the heart systole and diastole and can reflect physiological information regarding body components such as the atria, ventricles, and large vessels, as well as their functional states [3]. In general, fundamental heart sounds (FHSs) can be classified as the first heart sounds and the second heart sounds, referred to as S1 and S2, respectively. S1 usually occurs at the beginning of isovolumetric ventricular contraction, when the already closed mitral and tricuspid valves suddenly reach their elastic limit due to the rapid pressure increase within the ventricles. S2 occurs at the beginning of the diastole when the aortic and pulmonic valves close.

**Figure 1:**
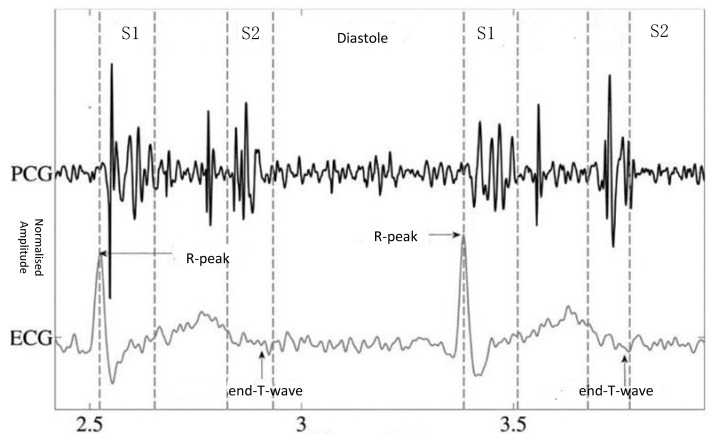
PCG with simultaneous ECG recording and the four states of the PCG recording: S1, the systole, S2, and the diastole [4].

It is important to segment the FHSs accurately and locate the state sequence of S1, the systole, S2, and the diastole. Figure 1 illustrates a PCG process with simultaneous electrocardiogram (ECG) recording and the four states of the PCG recording: S1, the systole, S2, and the diastole. The correspondence between the QRS waveform of the ECG and the heart sound signal is used to locate the S1 and S2 locations. FHSs provide important initial clues for heart disease evaluation in the process of further diagnostic examination. It is very important to extract the features from all parts of the FHS for quantitative analysis in the diagnosis of cardiac diseases. Within this framework, automatic heart sounds classification has attracted increased attention over the past few decades.

Achieving high accuracy in automatic heart sounds classification algorithms has long been a pursuit of researchers. Popular heart sound signals classification methods can be divided into two major categories: traditional machine learning-based methods and deep learning-based methods. With the recent development of medical big data and artificial intelligence technology, there has been increased focus on the development of deep learning methods for heart sounds classification [5]. However, despite the significant achievements in the field, there are still challenges that require the development of more robust methods with higher performance for early CVD diagnosis.

**Figure 2:**
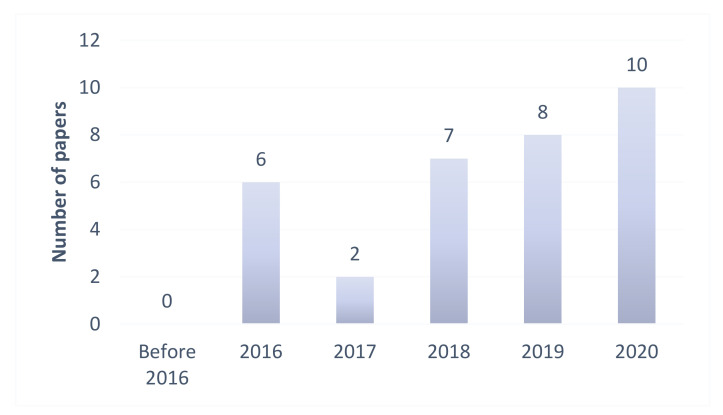
Previous studies on deep learning-based methods for heart sounds classification.

The purpose of the present study was to perform an in-depth and systematic review and analysis of the latest deep learning-based heart sounds classification methods and provide a reference for future research in the field. To this end, we used keywords such as heart sounds, PCG, deep learning, classification, neural network, and phonocardiogram to download relevant publications related to heart sounds classification from the databases of ScienceDirect, SpringerLink, IEEEXplore, and Web of Science. Thirty-three of the studies obtained in this manner were shortlisted and considered for review. To the best of our knowledge, these studies included all the essential contributions to the application of deep learning to heart sounds classification. These studies are summarized in Table 1, and some of them are discussed in more detail in this paper. Their distribution, including the numbers of articles and conference papers, is also shown in Figure 2. It was observed that most of the deep learning-based methods for heart sounds classification were published within the last three years, and that the number of published papers had drastically increased in the last five years, reflecting the increasing popularity of deep learning in the field. To the best of our knowledge, this is the first review report that consolidates the findings on deep learning technologies for heart sounds classification.

The remainder of this paper is organized as follows. Section 2 introduces the main process of heart sounds classification. Section 3 presents a consolidated review of different deep learning methods for heart sounds classification. Section 4 further discusses these methods, compares them with traditional machine learning methods, and examines the trends and challenges of the application of deep learning to heart sounds classification. The final conclusions are presented in Section 5.

## 2. Process of Heart Sounds Classification

**Figure 3:**
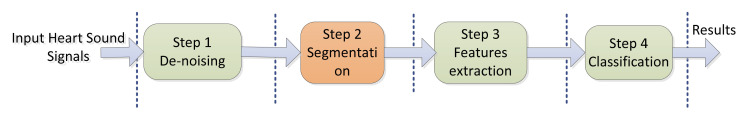
Four steps of automatic heart sounds classification.

As illustrated in Figure 3, the automatic heart sounds classification process generally consists of four steps: denoising, segmentation, feature extraction, and classification.

### 2.1. Denoising

The heart sounds acquisition process is easily affected by environmental interferences such as interference due to friction between the equipment and human skin, electromagnetic interference, and random noises such as breath sounds, lung sounds, and environment sounds [6]. The heart sound signals are usually coupled with these interference signals, and this necessitates the elimination of the out-of-band noise. The denoising significantly influences the segmentation, feature extraction, and final classification performances. The commonly used denoising methods are wavelet denoising, empirical mode decomposition denoising, and digital filter denoising [7]. Based on prior knowledge of heart sound signals, the construction of a wavelet basis function for heart sound signals is a new research direction in the area of heart sounds feature extraction [8].

### 2.2. Segmentation

The aim of the segmentation is the division of the PCG signals into four parts or segments: the first heart sounds (S1), systole, second heart sounds (S2), and diastole. Each segment contains efficient features that contribute to distinguishing the different categories of heart sounds. However, the duration of the heart beat cycle, the number of heart sounds, and the types of heart murmurs vary between individuals, and this causes the inaccurate segmentation of PCG signals. The segmentation of the FHSs is thus an essential step in automatic PCG analysis. The most commonly used heart sounds segmentation methods in recent years include envelope-based methods [9,10], ECG or carotid signal methods [11], probabilistic model methods [12,13,14,15], feature-based methods [16], and time–frequency analysis methods [17]. The utilized algorithms are based on the assumption that the diastolic period is longer than the systolic period. In fact, this assumption is not always true for an abnormal heart sound, especially in infants and cardiac patients [18]. Among these methods, those that utilize the cardiac cycle and an ECG signal, based on the correspondence between the ECG QRS waveform and the heart sound signals, have been found to yield better segmentation performance. However, their hardware and software requirements are greater. In addition, public heart sound databases rarely include synchronized ECG signals, which makes it difficult to segment the heart sound signals based on ECG signals.

### 2.3. Feature Extraction

Feature extraction is used to convert the raw high-dimensional heart sound signals into low-dimensional features through various mathematical transformations to facilitate the analysis of the heart sound signals. A variety of handcrafted features and machine learning-based methods have been applied for feature extraction, with the most common involving the use of Mel frequency cepstrum coefficients (MFCCs) [19,20], Mel domain filter coefficients (MFSCs), and heart sound spectra (spectrograms) [21], which are based on the short-time Fourier transform (STFT) and discrete wavelet transform (DWT) coefficients [18], and time and frequency features [22,23] from the time-domain, frequency-domain, and time–frequency or scale domain in the S1 and S2 components. The features extracted by STFT are difficult to balance with the time and frequency resolutions of the heart sound signals because the length of the window size impacts the resolution of the signals in both the time and frequency domains. Compared with these methods, the wavelet transform is more effective for the extraction of the main features of the heart sounds. Wavelet analysis has also been shown to afford high time and frequency resolutions and better representations of the S1 and S2 components [24].

### 2.4. Classification

Classification is used to divide the PCG signals into normal and abnormal categories. The utilized algorithms are of two main types: the first type of employed algorithms uses traditional machine learning methods such as artificial neural networks (ANNs), Gaussian mixture models, random forests, support vector machines (SVMs), and hidden Markov models (HMMs), which are applied to the extracted features to identify different heart sound signals symptomatic of different heart problems [5]; the other type of employed algorithms uses the latest popular deep learning methods such as deep CNNs and RNNs. In summary, although traditional machine learning methods have enabled significant achievements, they have limitations, which are detailed in Section 4.1.

## 3. Deep Learning for Heart Sounds Classification

**Figure 4:**
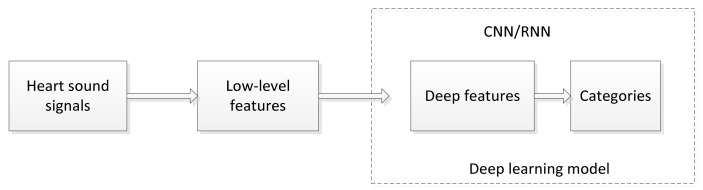
Process of heart sounds classification based on deep learning.

This section outlines the fundamental concepts and deep learning algorithms used for heart sounds classification. Figure 4 shows a typical block diagram of a deep learning approach to heart sounds classification. The approaches based on deep learning are mainly divided into CNN, RNN, and hybrid methods. Studies that describe these methods in detail are outlined in Table 1. Some of the details are presented in the following subsections, including the type of features that can be used as deep learning input vectors, applicable preprocessing techniques, and the process of constructing deep learning models for heart sounds classification.

**Table 1:** Deep learning-based methods for heart sounds classification.

S. No	Reference	Method	Input Features	Segment	Optimizer	Categories	Performance on Test Dataset MAcc, Se, Sp, Acc
**CNN-Based Methods**							
1	Maknickas et al., 2017 [25]	2D-CNN	MFSC	No	RMSprop	N, A	84.15, 80.63, 87.66, *
2	Tarik Alafif et al., 2020 [26]	2D-CNN + transfer learning	MFCC	NO	SGD	N, A	*, *, *, 89.5%
3	Deng et al., 2020 [24]	CNN + RNN	Improved MFCC	No	Adam	N, A	0.9834, 0.9866, 0.9801, *
4	Abduh et al., 2019 [27]	2D-DNN	MFSC	No	*	N, A	93.15, 89.30, 97.00, 95.50
5	Chen et al., 2018 [28]	2D-CNN	Wavelet transform + Hilbert–Huang features	No	*	N, M, EXT	93.25, 98, 88.5, 93
6	Rubin et al., 2016 [29]	2D-CNN	MFCC	Yes	Adam	N, A	83.99, 72.78, 95.21, *
7	Nilanon et al., 2016 [30]	2D-CNN	Spectrograms	No	SGD	N, A	81.11, 76.96, 85.27, *
8	Dominguez et al., 2018 [31]	2D-CNN	Spectrograms	No	*	N, A	94.16, 93.20, 95.12, 97.05
9	Bozkurt et al., 2018 [32]	2D-CNN	MFCC + MFSC	Yes	*	N, A	81.5, 84.5, 78.5, 81.5
10	Chen et al., 2019 [33]	2D-CNN	MFSC	No	Adam	N, A	94.81, 92.73, 96.90, *
11	Cheng et al., 2019 [34]	2D-CNN	Spectrograms	No	*	N, A	89.50, 91.00, 88.00, *
12	Fatih et al., 2019 [35]	2D-CNN	Spectrograms	No	*	N, M, EXT	0.80 (Accuracy on dataset A) 0.79 (Accuracy on dataset B)
13	Ryu et al., 2016 [36]	1D-CNN	1D time-series signals	No	SGD	N, A	78.69, 66.63, 87.75, *
14	Xu et al., 2018 [37]	1D-CNN	1D time-series signals	No	SGD	N, A	90.69, 86.21, 95.16, 93.28
15	Xiao et al., 2020 [38]	1D-CNN	1D time-series signals	No	SGD	N, A	90.51, 85.29, 95.73, 93.56
16	Humayun et al., 2020 [39]	tConv-CNN (1D-CNN)	1D time-series signals	Yes	Adam	N, A	81.49, 86.95, 76.02, *
17	Humayun et al., 2018 [40]	1D-CNN	1D time-series signals	Yes	SGD	N, A	87.10, 90.91, 83.29, *
18	Li et al., 2019 [41]	1D-CNN	Spectrograms	No	*	N, A	*, *, *, 96.48
19	Li et al., 2020 [42]	1D-CNN	497 features from time, amplitude, high-order statistics, cepstrum, frequency cyclostationary and entropy domains	Yes	Adam	N, A	*, 0.87, 0.721, 0.868
20	Xiao et al., 2020 [43]	1D-CNN	1D time-series signals	No		N, A	*, 0.86, 0.95, 0.93
21	Shu Lih Oh et al., 2020 [44]	1D-CNN WaveNet	1D time-series signals	NO	Adam	N, AS, MS, MR, MVP	0.953, 0.925, 0.981, 0.97
22	Baghel et al., 2020 [45]	1D-CNN	1D time-series signals	No	SGD	N, AS, MS, MR, MVP	*, *, *, 0.9860
**RNN-Based Methods**							
23	Latif et al., 2018 [46]	RNN (LSTM, BLSTM, GRU, BiGRU)	MFCC	Yes	*	N, A	98.33, 99.95, 96.71, 97.06 (LSTM) 98.61, 98.86, 98.36, 97.63 (BLSTM) 97.31, 96.69, 97.93, 95.42 (GRU) 97.87, 98.46, 97.28, 97.21 (BiGRU)
24	Khan et al., 2020 [47]	LSTM	MFCC	No	*	N, A	*, *, *, 91.39
25	Yang et al., 2016 [48]	RNN	1D time-series signals	No	*	N, A	80.18, 77.49, 82.87, *
26	Raza et al., 2018 [49]	LSTM	1D time-series signals	No	Adam	N, M, EXT	*, *, *, 80.80
27	Westhuizen et al., 2017 [50]	Bayesian LSTM LSTM	1D time-series signals	No		N, A	0.798, 0.707, 0.889, 0.798 0.7775, 0.675, 0.880, 0.778
**Hybrid Methods**							
28	Wu et al., 2019 [51]	Ensemble CNN	pectrograms + MFSC + MFCC	No	*	N, A	89.81, 91.73, 87.91, *
29	Noman et al., 2019 [52]	Ensemble CNN	(1D time-series signals + MFCC)	Yes	Scikit	N, A	88.15, 89.94, 86.35, 89.22
30	Tschannen et al., 2016 [53]	2D-CNN+SVM	Deep features	Yes	*	N, A	81.22, 84.82, 77.62, *
31	Potes et al., 2016 [23]	AdaBoost + 1D-CNN	Time and frequency features, MFCC	Yes	*	N, A	86.02, 94.24, 77.81, *
32	Gharehbaghi et al., 2019 [54]	STGNN + MTGNN	Time-series signal	No	*	N, A	*, 82.8, *, 84.2
33	Deperlioglu et al., 2020 [55]	AEN	1D time-series signals	No	*	N, M, EXT	0.9603 (Accuracy for normal), 0.9191 (Accuracy for extrasystole), 0.9011 (Accuracy for murmur)

* Abbreviations—N: normal heart sounds, M: murmur heart sounds, EXT: extrasystole heart sounds, AS: aortic stenosis, MS: mitral stenosis, MR: mitral regurgitation, MVP: mitral valve prolapse, MS: mitral stenosis, Acc: accuracy, MAcc: mean of specificity, Sp: specificity, Se: sensitivity.

### 3.1. CNN Methods for Heart Sounds Classification

**Figure 5:**
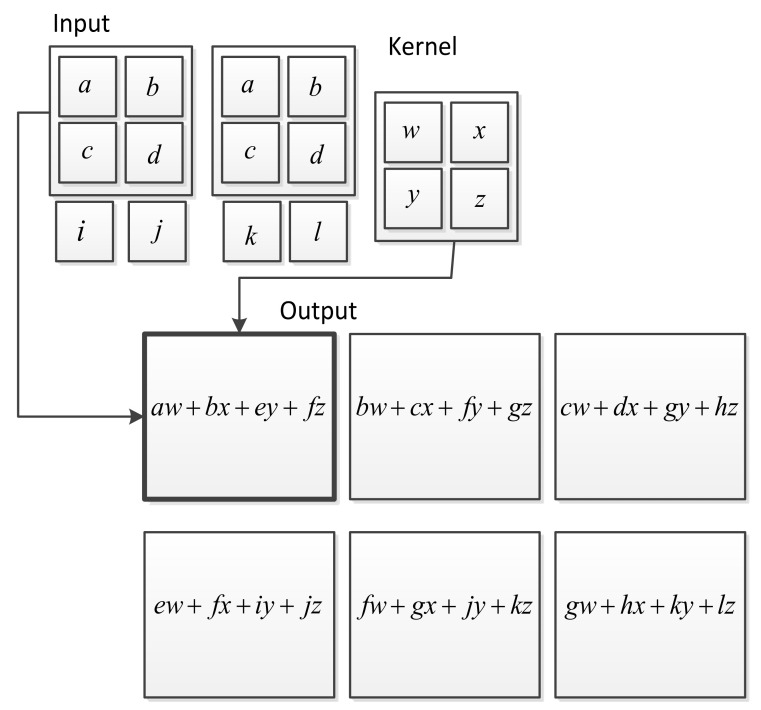
An example of 2D convolution operation in the architecture of CNNs [56].

A CNN, also known as a grid-like topology, is a specialized type of neural network for processing both time-series data and image data [56]. Figure 5 shows an example of convolution applied to a 2D tensor.

The kernel unit network of a CNN is a convolution network that uses a specialized kind of linear operation instead of general matrix multiplication in more than one layer. When the input is a one-dimensional vector, the out-feature map y  can be calculated by a discrete convolution operation, which is typically expressed as

$$y\left(n\right)=x\left(n\right)*\omega \left(n\right)=\overset{\infty }{\underset{m=-\infty }{\sum }}x\left(m\right)\omega \left(n-m\right)$$

where ∗ denotes a convolution operation and ω  is a convolution kernel. This is usually applied to a 1D CNN. In the case of a 2D CNN, the input x and the kernel ω are 2D matrixes, and the output feature map y can be computed as

$$Y\left(i,j\right)=X\left(i,j\right)*\omega \left(i,j\right)=\underset{m}{\sum }\underset{n}{\sum }X\left(m,n\right)\omega \left(i-m,j-n\right)$$

There are three important techniques for performing convolution operations: sparse interactions, equivariant representations, and parameter sharing [56]. Sparse connectivity is performed by making the kernel smaller than the input, with parameter sharing enabling more functions to use the same parameters in a model. This form of parameter sharing can particularly cause the layer to have a property referred to as equivariant representation. This specifically results in a function $f\left(x\right)$ being equivariant to a function $g\left(x\right)$ if $f\left(g\left(x\right)\right)=g\left(f\left(x\right)\right)$.

Two-dimensional CNN-based methods for heart sounds classification benefit from the successful application of CNNs to computer vision. Conventionally, the 1D heart sound signals are first converted into 2D feature maps that represent the time and frequency characteristics of the heart sound signals and satisfy the unified standards for 2D CNN inputs for heart sounds classification. The feature maps most commonly used for heart sounds classification include MFSC [19,25,32,33], MFCC [26,32], and spectrograms [30,31,34]. Rubin et al. [29] proposed a 2D CNN-based approach for the automatic recognition of normal and abnormal PCG signals. The heart sounds at the beginning of S1 were segmented into fixed 3 s segments using Springer’s segmentation algorithm [4]. These one-dimensional time series of the PCG signals were then converted into 2D feature maps using MFCCs, and the maps were utilized to train and validate the model. The method yielded an accuracy score of 72.78% in the 2016 PhysioNet/Computing in Cardiology (CinC) Challenge. Nilannon et al. [30] used a combination of MFCCs and spectrogram feature maps for feature extraction from fixed 5 s segments of PCG signals to train a CNN model with only one convolution layer. As a result, their best score was 81.1%, which is only 8.32% higher than the method in [29]. Maknickas et al. [25] developed a 2D CNN architecture and MFSCs for recognizing normal and abnormal PCG signals. The MFSCs were extracted from the log energies, which are obtained by STFT. The MFSC features were organized into three-channel feature maps, with the three channels representing the static features, first-order features, and second-order features of the MFSCs, respectively. The three-channel feature maps were fed into the 2D CNN model, which had a structure similar to that of an AlexNet network [57]. The network consisted of two fully connected layers, two convolutional layers, and two maximum pooling layers, with a total of 20,197,212 parameters. The method produced an average classification accuracy of 86.02%, achieving sixth place in the 2016 PhysioNet/CinC Challenge. In our previous study, we improved this method by using a combination of Inception [58] and Resnet [59] networks to develop a 138-layer CNN network [33]. The majority vote strategy was used to determine the category of the PCG signals, affording better robustness when applied to long heart sounds. In addition to MFSC features, MFCC features obtained by eliminating the inter-dimensional correlation through the discrete cosine transform (DCT) have also been utilized as the input vector of the CNN [24,26,29,32].

In 2D CNN-based methods, the convolution is performed in the time and frequency domains of the heart sound signals, and multiple levels of distributed representations are discovered from the low-level feature maps by the addition of more layers and more units within a layer. These feature maps represent both the time and frequency-domain features of the heart sound signals. However, the low-level features based on STFT are difficult to balance with the time and frequency resolutions of the heart sound signals because the length of the window size impacts the resolutions of the signals in both the time and frequency domains. Researchers choose an appropriate window size based on the assumption that the heart sound signals within the duration of the window size are stationary. Compared with STFT-based features, DWT-based features are more effective. Wavelet analysis has been shown to afford a better time resolution at higher frequencies and a lower frequency resolution at lower frequencies [24]. It also has a higher time–frequency resolution and enables better representations of the S1 and S2 components.

Although 2D feature maps provide good representations of acoustically significant patterns, they require an extra transform procedure and the use of a set of hyper-parameters. Furthermore, a 1D CNN, which is very effective for deriving features from shorter 1D signals and when the location of the feature within the segment is not very important, can be used to construct a deep learning model for heart sounds classification. Consequently, various 1D CNN-based methods with different CNN architectures have been proposed for identifying different kinds of heart sounds [36,37,38,39,40,41,60]. In a typical example, the 1D PCG time series is directly used as the 1D CNN without any spatial domain transform such as STFT or DWT. Xu et al. [37] proposed a novel 1D deep CNN for PCG patch classification. The CNN had a block-stacked style architecture and fewer parameters and used bidirectional connections to enhance the information flow. The method achieved the highest accuracy score of 90.046% in the 2016 PhysioNet/CinC Challenge, based on only 0.19 M trainable parameters, which is 1/65 of that for the 2D CNN [29]. Nevertheless, the 1D CNN-based method provided a heart sound classification performance comparable with that of the 2D CNN without the requirement for feature engineering. It is designed to efficiently reuse the feature maps with less parameter consumption and without extra preprocessing. One-dimensional CNN-based methods usually perform the task without complex preprocessing and the use of the numerous hyper-parameters that are required by 2D CNN-based methods.

### 3.2. RNN Methods for Heart Sounds Classification

RNNs are a family of neural networks specifically used for processing sequential data. RNN architectures such as gated recurrent units (GRUs) and long short-term memory (LSTM) have been reported to deliver state-of-the-art performances in numerous applications, including machine translation, speech recognition, and image captioning [56].

Heart sound signals are a kind of sequential data with a strong temporal correlation and can thus be suitably processed by RNNs. Indeed, they have been proven to be very effective and are commonly used for heart sounds classification [46,47,48,49,50].

**Figure 6:**
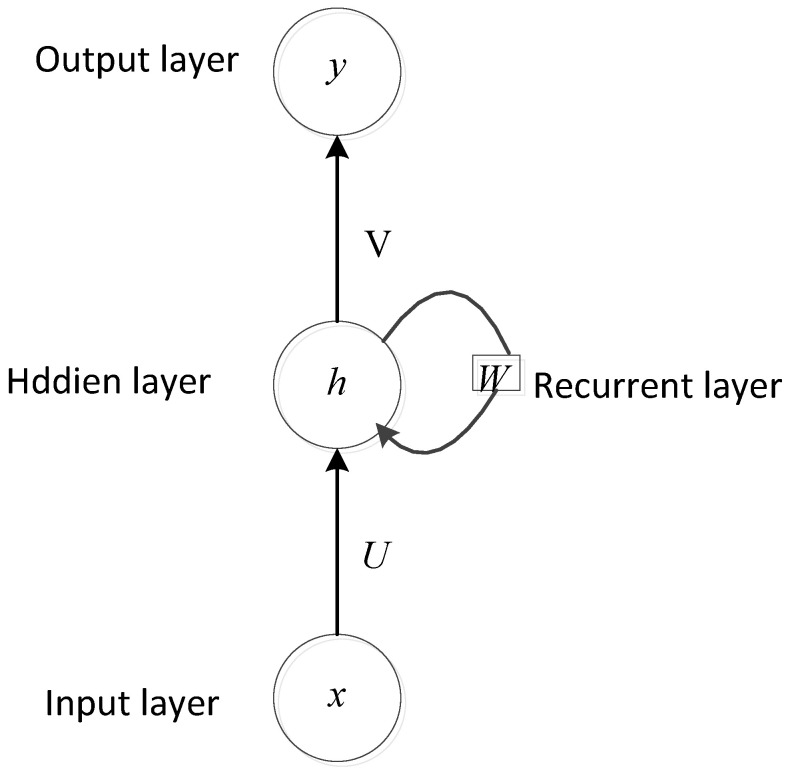
General diagram of an RNN architecture [56].

In the application of an RNN-based heart sounds analysis method, the RNN accepts an input in the form of a 1D heart sound signal $x\left(t\right)=\left(x_{1},\ldots ,x_{T}\right)$ and, at the current time t, computes the hidden information or memory of the network, $h_{t}$, using the previous state $h_{t-1}$ and the input signal $\;x_{t}$. The softmax function is utilized to project the output vector onto the probability corresponding to the number of heart sound classes. The standard equations used are

$$h_{t}=H\left(U\cdot x_{t}+W\cdot h_{t-1}+b\right)\;y_{t}=softmax\left(V\cdot h_{t}+c\right)$$

where U, V, and W are the weight matries, *H* is the hidden layer function, and *b*, *c* are the bias vectors. In Figure 6, a general diagram of an RNN architecture is presented.

The key pattern for an RNN is that it can make an output at each time step and have recurrent connections between the output at one time step and the hidden units at the next time step, and these connections of the RNN can read an entire sequence and then yield a single output. To avoid the problem of vanishing and exploding gradients in RNNs, a gated RNN based on the idea of creating paths through time is usually used to control the speed of information accumulation. Meanwhile, a standard RNN has a limitation; namely, that it can only use previous information to make decisions. A bidirectional RNN with bidirectional long short-term memory (BLSTM) and bidirectional gated recurrent units (BiGRUs), which use LSTM units or GRUs, can be used for both forward and backward information processing, enabling the exploitation of future contexts [61]. All the above RNN variants have been used in heart sounds classification.

Yang and Hsieh [48] were the first to use an RNN-based method to detect anomalies in heart sounds provided by the 2016 PhysioNet/CinC Challenge. They utilized a deep learning model consisting of four layers: the first layer is a GRU with 386 features; the second layer is a dropout layer; the third layer is a GRU with eight features; and the last layer is a fully connected layer. Because of the shallowness of the network and the limited training data, it only achieved a total score of 80.18%. However, compared with other non-deep learning methods, it eliminates the tedious process of manual feature extraction. Khan et al. [47] used LSTM in combination with the MFCC features of the unsegmented data and achieved the best area under the curve score of 91.39% in the PhysioNet/CinC Challenge, performing better than other algorithms such as SVM, KNN, decision tree, and ANN for various time and frequency-domain features. Similarly, Raza et al. [49] used the LSTM model to classify three kinds of heart sounds—namely, normal, murmur, and extrasystole—in PASCAL heart sound dataset B. As a result, an accuracy of 80.80% was achieved by this method. In contrast, the method presented in [48] uses a band-pass filter to remove the noise in the heart sound signals during normalized preprocessing and then uses a down-sampling technique to reduce the dimension of the data and further fix the dimension of the heart sound signals by truncation and copying. This further illustrates the advantages of RNN compared with the traditional classifier for heart sounds classification and recognition. Furthermore, Latif et al. [46] examined four different types of RNNs; namely, LSTMs, BLSTMs, GRUs, and BiGRUs. The heart sound data were segmented into five cardiac cycles using the HSMM method [15], and the MFCC features were extracted for use as the input of the RNN models. The best classification performance evaluated on the dataset of the 2016 PhysioNet/CinC Challenge was found to be obtained by using two gating layers in the cyclic neural network model. The total accuracy of the BLSTM for normal and abnormal recognition was as high as 97.63%, which was 11.61%, 14.83%, 13.64%, and 3.47% higher than those of Potes [23], Tschannen [53], Rubin [29], and Dominguez [31], which were based on CNN. Westhuizen et al. [50] proposed a Bayesian LSTM model with two hidden layers containing 128 units and used dropout for the classification of heart sounds. The model was applied to the dataset of the 2016 PhysioNet/CinC Challenge and achieved an accuracy 2% higher than that of the standard LSTM.

As mentioned above, both 1D CNNs and RNNs can be used to analyze time-series heart sound signals for classification tasks with greater accuracy than traditional algorithms. RNN differs from 1D convolution in that each member of its output produced by the same update rule applied to the previous outputs is a function of the previous members of the output, resulting in the sharing of parameters through a very deep computational graph. However, 1D convolution allows a network to share parameters across time and is thus very shallow. In particular, RNNs are able to process sequential signals of deep length by using neurons with self-feedback; nevertheless, LSTMs have poor performance on signals longer than 1000 time steps [62].

### 3.3. Hybrid Methods for Heart Sounds Classification

Various integrations of different deep learning models for heart sounds classification have been proposed in recent years. The integrated methods are mainly divided into model-based and feature-based types. Some hybrid deep learning networks have also been developed. The most typical model-based integrated methods combine CNNs and RNNs. There are two reasons for this particular combination. The first is that CNNs use various stacked convolution kernels to extract the features layer-by-layer, with the different convolution kernels capturing different kinds of features. Compared with a fully connected neural network, CNNs improve the efficiency of feature extraction and significantly reduce the amount of required computation. The second reason is that RNNs use the loop unit as the core of the structure, with each unit receiving the data of the current and previous time steps as its input. This increases the correlation between two successive time steps, resulting in an RNN having the advantages of being able to process the signals of the timing relationship. Therefore, the fusion of CNN and RNN models thus results in a mutual complement. Deng et al. [24] exploited the spatial and temporal characteristics extracted from the CNN and RNN, respectively, to achieve a higher accuracy. Both CNN and RNN have strong feature extraction capabilities that enable the direct classification of normal and abnormal PCGs from the original data, eliminating the need for complex segmentation of the heart sound features. The extraction procedure fully utilizes the global characteristics of the heart sound data and facilitates the simultaneous extraction of the frequency and time-domain information, resulting in better performance than a single model.

A 1D CNN has also been combined with a 2D CNN to develop a TF-ECNN deep learning model for learning multiple levels of representations [52]. The 1D CNN was designed to learn the time-domain features from raw PCG signals, while 2D CNN learned the time–frequency features. This combination achieved an accuracy of 89.22%, which is 2.88% higher than that of the 1D CNN, and 2.81% higher than that of the 2D CNN. Potes et al. utilized an ensemble of AdaBoost and CNN classifiers to classify normal and abnormal heart sounds [23]. They used 124 time–frequency features to train the first AdaBoost classifier and decomposed the PCG cardiac cycles into four frequency bands to train the second CNN classifier. Both the time and frequency-domain features were extracted from the segmented PCG signals. This ensemble approach yielded the highest performance in the PhysioNet/CinC Challenge. Noman et al. [52] also developed a framework based on a 1D CNN and 2D CNN in which short segments of heart beats were used for PCG classification. The 1D CNN was designed to learn features from the raw datasets, and 2D time–frequency feature maps were inputted to the 2D CNN. This 1D and 2D CNN combination yielded a classification accuracy score of 89.22%, which is significantly higher than those of traditional SVM and HMM-based classifiers. Ensemble models are thus superior to traditional and individual DL models, and they also solve the over-fitting problem. However, they require more computing resources and their use is time-consuming.

In data-based methods, heart sounds are classified by combining various features and using different classifiers. For example, Tschannen et al. [53] proposed a robust feature representation based on spectral and deep features extracted by CNNs, with an SVM used as a classifier. The method yielded a score of 81.2%, which is better than that achieved using only wavelet transform features. In [51], a Savitzky Golay filter was used to denoise the heart sounds, and three features—namely, the spectrogram, MFSC, and MFCC—were then used as the input for multiple CNNs combined by three modified VGGNets. An average accuracy of 89.81% was achieved in 10-fold cross-validation experiments, representing an improvement of 5.98%, 3%, and 5.52% compared with the individual uses of the spectrogram, MFSC, and MFCC, respectively, to train the models. Thus, the combined use of model-based and feature-based methods produced more accurate classification results compared with the use of only a single deep learning model. However, this was also at the cost of more computing resources and time.

As can be observed from Table 1, most of the reviewed studies involved two-class—i.e., normal and abnormal—heart sounds classification. Due to the scarcity of the required datasets, only a few studies considered more classification classes [28,35,44,45,49,55]. Fatih et al. [35] combined three pre-trained deep learning models—namely, VGG16, VGG19, and AlexNet—for the classification of heart sounds into three types—namely, normal (N), murmur (M), and extrasystole (EXT)—in the PASCAL Classifying Heart Sound Challenge [63]. However, despite significantly improving the classification accuracy compared with the baseline method, this ensemble method requires considerable time for training and prediction using the classifiers. Shu Lih Oh et al. [44] proposed a deep learning model, WaveNet, consisting of six residual blocks for the classification of five types of heart sounds—normal, aortic stenosis, mitral regurgitation, mitral stenosis, and mitral valve prolapse—that are available in public databases [64]. The method produced a high training accuracy of 97% before the development of the Fatih method [35]. It should be noted that the utilized datasets were balanced, with each class of heart sound containing 200 recordings. Most deep learning-based methods do not utilize a segmentation algorithm to identify S1, S2, systole, and diastole heart sounds, such as [25,26,27,28,30,31,33,34,35,36,37,38,44,45,47,48,49,50,51,54,55]. The methods are nevertheless very efficient for automatic heart sounds classification.

## 4. Discussion

The strengths and limitations of heart sounds classification methods based on deep learning and traditional machine learning methods and the trends and challenges of these methods are discussed in this section.

### 4.1. Comparison of Deep Learning and Traditional Machine Learning Methods

**Figure 7:**
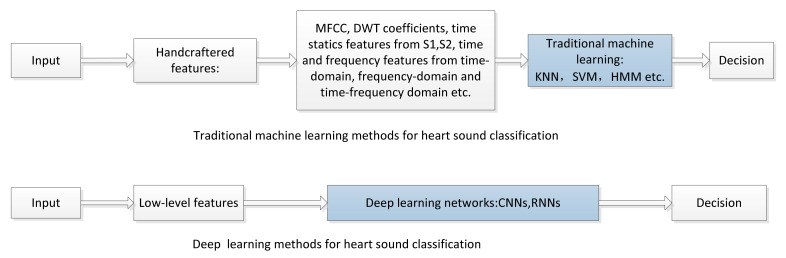
Different processes of traditional machine learning and deep learning methods for heart sounds classification.

Deep learning enables the automatic extraction of the sound characteristics from the raw data signals and the determination of the rules among the data. Compared with traditional machine learning approaches, deep learning approaches are more efficient and accurate. The different processes of traditional machine learning approaches and deep learning approaches for heart sounds classification are shown in Figure 7. According to a survey [5], the accuracy of heart sounds classification based on deep learning is generally higher than that based on traditional machine learning. The strengths and limitations of deep learning and traditional machine learning for heart sounds classification are summarized in Table 2.

In most studies on traditional machine learning methods for heart sounds classification, a segmentation algorithm was used to identify the locations of the S1, S2, systole, and diastolic phases. Based on these locations, the time-domain, frequency-domain, and statistical features were extracted from the segmented heart sounds. The typical traditional machine learning approach for the automatic segmentation and classification of heart sounds was proposed by Pedro Narváez [65]. In this work, the empirical wavelet transform (EWT) and the normalized average Shannon energy (NASE) were used to automatically identify cardiac cycles and locate the segments of the S1, systole, S2 and diastole in a recording. This method has given better results than other machine learning methods such as discrete wavelet transform, Butterworth or Chebyshev filters and empirical mode decomposition (EMD). However, in the segmented heart sounds classification, the process of heart sounds classification is more complicated and increases the complexity of the computation. Conversely, in unsegmented heart sounds classification, a small segment of the heart sounds is directly converted into representation features, without the need of the computational cost for substantial feature engineering. The classification performance is also comparable with that of methods that utilize heart sounds segmentation.

Traditional machine learning methods for heart sounds classification generally use small-scale training data, and the feature learning is based on prior knowledge of the data. They thus mostly rely on learned distributed discriminative features. However, the essence of deep learning is to build a neural network with multiple hidden layers and to hierarchically fine-tune all the model parameters from the bottom to the top through massive data training. Strong generalized and abstraction features are extracted step-by-step from the low-level features of the raw data, and the prediction is made easier through the use of end-to-end networks, resulting in improved classification accuracy. Unlike traditional machine learning methods, the single architecture of a deep learning method can be used for joint feature extraction, feature selection, and classification. Deep learning is thus very effective for heart sounds classification while eliminating the need for the complicated feature engineering required by traditional machine learning.

### 4.2. Trends and Challenges

#### 4.2.1. Training with Limited Heart Sound Data

Deep learning-based methods for heart sounds classification require a large amount of widely distributed heart sound data to avoid over-fitting and enhance and broaden the performance of the trained model. It is widely believed that extending training heart sound datasets and DL-based heart sounds classification models should improve accuracy and result in better performance. In practice, the amount of heart sound data we have is limited. Consequently, determining the minimum number of training heart sound samples needed to achieve high accuracy for heart sounds classification is imperative. However, to the best of our knowledge, there are no existing works that discuss this issue. We can consider [66], in which a general methodology is presented that can be easily applied to this issue to generate learning curves and determine the necessary heart sound dataset sizes. Furthermore, to advance the state-of-the-art, the relationships between training set size, computational scale, and model accuracy improvements should be understood.

In addition, the deep learning performances of most of the utilized algorithms are usually benchmarked using datasets provided in the 2016 PhysioNet/CinC Challenge. However, such widely used publicly available heart sound datasets are of small sizes, especially with regard to specific diseases, and these available datasets typically only include the sound waveform, excluding relevant clinical information such as gender, age, and history of illness, which is very important for doctors to perform their assessment. However, it is time-consuming and labor-consuming to acquire a large amount of heart sound samples, especially for a specific type of abnormality. Therefore, existing public heart sound datasets are usually scarce and imbalanced among different classes. This brings great challenges in classifying these sounds accurately in a real clinical application when using deep learning technology.

Besides, most existing deep learning methods have concentrated on the two-class (normal and abnormal) problems of heart sounds classification. However, due to the limited heart sound data, a few studies have researched the classification of heart sounds with more classes. In the future, it will be necessary to collaborate with doctors to build standard heart sound databases that can record clinical information such as gender, age, position, and history of illness, et al., and share the databases on a cloud platform. This may allow deep learning methods to identify more specific anomalies in heart sound signals.

Some technologies such as batch normalization, regularization, and dropout can be used to avoid over-fitting in the training of deep models and maximize the generalization performance; the variety of recording equipment, environmental noise, and collection locations involved in the acquisition of heart sound signals directly lead to diversity in the data distribution. It was shown in [39] that even the best model trained on the PhysioNet/CinC Challenge datasets [23] achieved only 50.25% accuracy when tested on the HSSDB dataset. This is because of the limited available heart sound data, resulting in over-fitting and low accuracy when applied to deep learning methods. There are currently three approaches to addressing the need for an enormous amount of training data.

The first approach is to augment and balance the available data through various signal processing techniques such as oversampling and down-sampling [25,42,45,67]. Maknickas et al. [25] used an oversampling scheme to augment small heart sound samples and balance the positive and negative samples and were able to effectively improve the performance of classifying positive and negative samples. In addition, the use of synthetic heart sound data has become an effective augmentation method. Thomae et al. [68] used various audio effects such as tempo, speed, volume, and pitch to artificially increase their amount of training data, specifically increasing the raw heart sound recordings from 3153 to 53,601. This effectively prevented memorization and improved generalization. Baghel et al. [45] used the background deformation technique for the augmentation to improve the performance in a noisy environment.

The second approach to solving the data limitation problem involves the modification of the algorithms by applying different weights to the cost function based on the distribution of the training data. This addresses the issues of imbalances in the classification of heart sounds. The distribution is biased to the high-cost classes and the model gives more attention to the samples with a small amount of heart sound data. This method has been widely applied in the fields of speech and image classification. However, there are only a few related works, such as [42], in which the optimization of the imbalance problem in the classification of the heart sounds has been used to improve the accuracy of the deep learning model, offering an ideal research direction.

Besides, generative adversary networks (GANs), a kind of architecture of deep neural networks consisting of two neural networks called the generator and discriminator, have been widely explored in the generation of synthetic images and speech. A GAN-based method has also been applied in the fields of heart sounds classification. For instance, Narváez, P. et al. [69] proposed a GAN-based model accompanied by a denoising stage using the EWT in order to generate synthetic heart sounds with a low noise level. GAN-based synthetic heart sounds are able to output varied synthetic heart sounds that are indistinguishable from natural sounds and augment existing databases to train deep learning models. As a result, the synthetic heart sounds can be used improve the performance of heart sounds classification models.

#### 4.2.2. Training Efficiency

A central challenge in deep learning is achieving efficient model training. It is often inefficient to train deep learning neural networks from scratch through the random initialization of the parameters. Indeed, having a large number of factors significantly affects the success of the training, with the most important aspects including the learning rate, optimizer, iteration step, and activation function. It is common to explore these optimization super-parameters through repeated experiments; however, this makes the training process extremely time-consuming. Training the deep learning model to automatically select the super-parameters remains a big challenge. A proper solution would involve the application of a transfer learning technique.

Compared with training from scratch, transfer learning can be used to accelerate training and achieve better results. The technique has been used in other fields such as acoustic classification paradigms, image classification, and natural language processing but has been rarely applied to heart sounds classification. In [68,70], the authors demonstrate the efficiency of training a deep model by transfer learning. However, Ren et al. [70] were the first to explore the application of transfer learning to heart sounds classification. They replaced the last fully connected layer of the model with two neurons by adapting the parameters of VGG16 to the heart sound data. This represents a faster means of achieving a full CNN-based classification compared with training the entire CNNs from scratch and has been reported to afford a significant improvement of 19.8% relative to the baseline [70].

Alafif et al. [26] used transfer learning to automate the recognition of normal and abnormal heart sounds. They used the MFCC representation as the input to various pre-trained CNN models such as SqueezeNet, GoogLeNet, Inception-V3, and Xception, which were fine-tuned on the new dataset. This approach is effective for training deep learning models and was used to achieve an average classification accuracy as high as 89.5% on the PASCAL Heart Sound Challenge dataset.

In addition, Humayun et al. [71] proposed a 1D CNN in which transfer learning was used to learn the parameters of a 1D CNN model pre-trained on the PhysioNet HS Classification dataset. The flattened layer was transferred [72] to a new CNN architecture with a fully connected layer and three output neurons representing normal, mildly abnormal, and severely abnormal categories, respectively. The parameters were fine-tuned on TL-Data, which are different from the samples in the PhysioNet heart sounds classification dataset. This process enables the avoidance of tedious super-parameter exploration and accelerates the model training process.

#### 4.2.3. More Powerful Models

Deep learning models with deeper layers normally exhibit more accurate performance, and this has been the tendency in recent developments. Examples of such models are the modified AlexNet network [31] with 35 convolutional layers, the modified VGGNet network [51] with 16 convolutional layers, and the modified InceptionResNet network [33] with 138 convolutional layers. These models achieved heart sounds classification accuracies of 97.05%, 93.56%, and 89.81%, respectively, which are significantly higher than those of models with only two [29] or one [30] convolutional layer. However, deep learning models with deeper layers may be characterized by higher system complexity, requiring a larger memory and more computing resources. This would significantly limit their application in mobile devices and other systems.

There are several directions that can be employed with the aim of developing efficient light-weight deep learning models. One involves the compression of the model by reducing the amount of redundant weights in the DNNs, thereby decreasing the memory and computing resource demands. The major current methods for compressing deep learning models include parameter pruning and sharing, low-rank factor decomposition, knowledge distillation, sparse regularization, and mask acceleration [56]. The application of such methods to deep learning models for heart sound signals classification would effectively reduce the storage space requirement and increase the computing speed of the adaptation to mobile terminal operations.

## 5. Conclusions

CVDs incur a heavy burden on human health and personal finances, especially in low and middle-income economies. Because heart sounds provide important initial clues for the evaluation of the condition of the human heart, computer-aided techniques for the quantitative analysis and classification of heart sounds can be used to facilitate the early diagnosis of CVDs for further examination. Many deep learning techniques for heart sounds classification have been developed in recent years. Deep learning has emerged as an ideal approach for the classification of heart sounds corresponding to different pathological conditions of the heart. Despite the advancements in the field, there are still limitations that necessitate the further development of the technology. The major problems requiring solutions include data insufficiency, training inefficiency, and insufficiently powerful models. The development of solutions for these challenges promises to make deep learning a major breakthrough for human health management.

## Figures and Tables

**Table 2:** Strengths and limitations of deep learning and traditional machine learning methods for heart sounds classification.

Approaches	Strengths	Limitations
Tradition machine learning	1. Easy to train. 2. Can effectively and quickly solve the objective function by convex optimization algorithm.	1. Has a complex data preprocess and the segmenting of heart sound signal is indispensable. 2. Has generalization and robustness issues.
Deep learning	1. Can effectively and automatically learn feature representations and the trained model is very good generally. 2. Good performance in classification.	1. The training process takes a long-time and is affected by limited datasets. 2. High requirements for hardware configuration.

## References

[1] WHO Cardiovascular Diseases (CVDs) [EB/OL]. https://www.who.int/zh/news-room/fact-sheets/detail/cardiovascular-diseases-(cvds).

[2] Liu C., Springer D., Li Q., Moody B., Juan R.A., Chorro F.J., Castells F., Roig J.M., Silva I., Johnson A.E.W. (2016). An open access database for the evaluation of heart sound algorithms. Physiol. Meas..

[3] Liu C., Murray A. (2017). Applications of Complexity Analysis in Clinical Heart Failure. Complexity and Nonlinearity in Cardiovascular Signals.

[4] Springer D.B., Tarassenko L., Clifford G.D. (2016). Logistic Regression-HSMM-Based Heart Sound Segmentation. IEEE Trans. Biomed. Eng..

[5] Dwivedi A.K., Imtiaz S.A., Rodriguez-Villegas E. (2019). Algorithms for Automatic Analysis and Classification of Heart Sounds—A Systematic Review. IEEE Access.

[6] Li S., Li F., Tang S., Xiong W. (2020). A Review of Computer-Aided Heart Sound Detection Techniques. BioMed Res. Int..

[7] Thalmayer A., Zeising S., Fischer G., Kirchner J. (2020). A Robust and Real-Time Capable Envelope-Based Algorithm for Heart Sound Classification: Validation under Different Physiological Conditions. Sensors.

[8] Kapen P.T., Youssoufa M., Kouam S.U.K., Foutse M., Tchamda A.R., Tchuen G. (2020). Phonocardiogram: A robust algorithm for generating synthetic signals and comparison with real life ones. Biomed. Signal Process. Control.

[9] Giordano N., Knaflitz M. (2019). A Novel Method for Measuring the Timing of Heart Sound Components through Digital Phonocardiography. Sensors.

[10] Wei W., Zhan G., Wang X., Zhang P., Yan Y. A Novel Method for Automatic Heart Murmur Diagnosis Using Phonocardiogram. Proceedings of the 2019 International Conference on Artificial Intelligence and Advanced Manufacturing, AIAM.

[11] Malarvili M., Kamarulafizam I., Hussain S., Helmi D. (2003). Heart sound segmentation algorithm based on instantaneous energy of electrocardiogram. Comput. Cardiol..

[12] Oliveira J.H., Renna F., Mantadelis T., Coimbra M.T. (2018). Adaptive Sojourn Time HSMM for Heart Sound Segmentation. IEEE J. Biomed. Health Inform..

[13] Kamson A.P., Sharma L., Dandapat S. (2019). Multi-centroid diastolic duration distribution based HSMM for heart sound segmentation. Biomed. Signal Process. Control..

[14] Renna F., Oliveira J.H., Coimbra M.T. (2019). Deep Convolutional Neural Networks for Heart Sound Segmentation. IEEE J. Biomed. Health Inform..

[15] Liu C., Springer D., Clifford G.D. (2017). Performance of an open-source heart sound segmentation algorithm on eight independent databases. Physiol. Meas..

[16] Chen T.E., Yang S.I., Ho L.T., Tsai K.H., Chen Y.H., Chang Y.F., Wu C.C. (2017). S1 and S2 heart sound recognition using deep neural networks. IEEE Trans. Biomed. Eng..

[17] Liu Q., Wu X., Ma X. (2018). An automatic segmentation method for heart sounds. Biomed. Eng. Online.

[18] Deng S.-W., Han J.-Q. (2016). Towards heart sound classification without segmentation via autocorrelation feature and diffusion maps. Future Gener. Comput. Syst..

[19] Abduh Z., Nehary E.A., Wahed M.A., Kadah Y.M. (2019). Classification of Heart Sounds Using Fractional Fourier Transform Based Mel-Frequency Spectral Coefficients and Stacked Autoencoder Deep Neural Network. J. Med. Imaging Health Inf..

[20] Nogueira D.M., Ferreira C.A., Gomes E.F., Jorge A.M. (2019). Classifying Heart Sounds Using Images of Motifs, MFCC and Temporal Features. J. Med Syst..

[21] Soeta Y., Bito Y. (2015). Detection of features of prosthetic cardiac valve sound by spectrogram analysis. Appl. Acoust..

[22] Chakir F., Jilbab A., Nacir C., Hammouch A. (2018). Phonocardiogram signals processing approach for PASCAL Classifying Heart Sounds Challenge. Signal Image Video Process..

[23] Potes C., Parvaneh S., Rahman A., Conroy B. (2016). Ensemble of feature based and deep learning-based classifiers for detection of abnormal heart sounds. Proc. Comput. Cardiol. Conf..

[24] Deng M., Meng T., Cao J., Wang S., Zhang J., Fan H. (2020). Heart sound classification based on improved MFCC features and convolutional recurrent neural networks. Neural Netw..

[25] Maknickas V., Maknickas A. (2017). Recognition of normal abnormal phonocardiographic signals using deep convolutional neural networks and mel-frequency spectral coefcients. Physiol. Meas..

[26] Alafif T., Boulares M., Barnawi A., Alafif T., Althobaiti H., Alferaidi A. Normal and Abnormal Heart Rates Recognition Using Transfer Learning. Proceedings of the 2020 12th International Conference on Knowledge and Systems Engineering (KSE).

[27] Abduh Z., Nehary E.A., Wahed M.A., Kadah Y.M. (2019). Classification of heart sounds using fractional fourier transform based mel-frequency spectral coefficients and traditional classifiers. Biomed. Signal Process. Control.

[28] Chen L., Ren J., Hao Y., Hu X. (2018). The Diagnosis for the Extrasystole Heart Sound Signals Based on the Deep Learning. J. Med. Imaging Health Inform..

[29] Rubin J., Abreu R., Ganguli A., Nelaturi S., Matei I., Sricharan K. Classifying heart sound recordings using deep convolutional neural networks and mel-frequency cepstral coefficients. Proceedings of the 2016 Computing in Cardiology Conference (CinC).

[30] Nilanon T., Yao J., Hao J., Purushotham S. Normal/abnormal heart sound recordings classification using convolutional neural network. Proceedings of the Computing in Cardiology Conference (CinC).

[31] Dominguez-Morales J.P., Jimenez-Fernandez A.F., Dominguez-Morales M.J., Jimenez-Moreno G. (2018). Deep Neural Networks for the Recognition and Classification of Heart Murmurs Using Neuromorphic Auditory Sensors. IEEE Trans. Biomed. Circuits Syst..

[32] Bozkurt B., Germanakis I., Stylianou Y. (2018). A study of time-frequency features for CNN-based automatic heart sound classification for pathology detection. Comput. Biol. Med..

[33] Chen W., Sun Q., Wang J., Wu H., Zhou H., Li H., Shen H., Xu C. (2019). Phonocardiogram Classification Using Deep Convolutional Neural Networks with Majority Vote Strategy. J. Med. Imaging Health Inform..

[34] Cheng X., Huang J., Li Y., Gui G. (2019). Design and Application of a Laconic Heart Sound Neural Network. IEEE Access.

[35] Demir F., Şengür A., Bajaj V., Polat K. (2019). Towards the classification of heart sounds based on convolutional deep neural network. Health Inf. Sci. Syst..

[36] Ryu H., Park J., Shin H. Classification of heart sound recordings using convolution neural network. Proceedings of the 2016 Computing in Cardiology Conference (CinC).

[37] Xu Y., Xiao B., Bi X., Li W., Zhang J., Ma X. Pay more attention with fewer parameters: A novel 1-D convolutional neural network for heart sounds classification. Proceedings of the Computing in Cardiology Conference (CinC).

[38] Xiao B., Xu Y., Bi X., Li W., Ma Z., Zhang J., Ma X. (2020). Follow the Sound of Children’s Heart: A Deep-Learning-Based Computer-Aided Pediatric CHDs Diagnosis System. IEEE Internet Things J..

[39] Humayun A.I., Ghaffarzadegan S., Ansari I., Feng Z., Hasan T. (2020). Towards Domain Invariant Heart Sound Abnormality Detection Using Learnable Filterbanks. IEEE J. Biomed. Health Inform..

[40] Humayun A.I., Ghaffarzadegan S., Feng Z., Hasan T. Learning front-end filter-bank parameters using convolutional neural networks for abnormal heart sound detection. Proceedings of the 2018 40th Annual International Conference of the IEEE Engineering in Medicine and Biology Society (EMBC).

[41] Li F., Liu M., Zhao Y., Kong L., Dong L., Liu X., Hui M. (2019). Feature extraction and classification of heart sound using 1D convolutional neural networks. EURASIP J. Adv. Signal Process..

[42] Li F., Tang H., Shang S., Mathiak K., Cong F. (2020). Classification of Heart Sounds Using Convolutional Neural Network. Appl. Sci..

[43] Xiao B., Xu Y., Bi X., Zhang J., Ma X. (2020). Heart sounds classification using a novel 1-D convolutional neural network with extremely low parameter consumption. Neurocomputing.

[44] Oh S.L., Jahmunah V., Ooi C.P., Tan R.-S., Ciaccio E.J., Yamakawa T., Tanabe M., Kobayashi M., Acharya U.R. (2020). Classification of heart sound signals using a novel deep WaveNet model. Comput. Methods Programs Biomed..

[45] Baghel N., Dutta M.K., Burget R. (2020). Automatic diagnosis of multiple cardiac diseases from PCG signals using convolutional neural network. Comput. Methods Programs Biomed..

[46] Latif S., Usman M., Rana R., Qadir J. (2018). Phonocardiographic sensing using deep learning for abnormal heartbeat detection. IEEE Sens. J..

[47] Khan F.A., Abid A., Khan M.S. (2020). Automatic heart sound classification from segmented/unsegmented phonocardiogram signals using time and frequency features. Physiol. Meas..

[48] Yang T.-C., Hsieh H. Classification of acoustic physiological signals based on deep learning neural networks with augmented features. Proceedings of the 2016 Computing in Cardiology Conference (CinC).

[49] Raza A., Mehmood A., Ullah S., Ahmad M., Choi G.S., On B.W. (2019). Heartbeat sound signal classification using deep Learning. Sensors.

[50] Van der Westhuizen J., Lasenby J. (2017). Bayesian LSTMs in Medicine, Unpublished Paper. https://arxiv.org/abs/1706.01242.

[51] Wu J.M.-T., Tsai M.-H., Huang Y.Z., Islam S.H., Hassan M.M., Alelaiwi A., Fortino G. (2019). Applying an ensemble convolutional neural network with Savitzky–Golay filter to construct a phonocardiogram prediction model. Appl. Soft Comput..

[52] Noman F., Ting C.-M., Salleh S.-H., Ombao H. Short-segment heart sound classification Using an ensemble of deep convolutional neural networks. Proceedings of the ICASSP 2019—2019 IEEE International Conference on Acoustics, Speech and Signal Processing (ICASSP).

[53] Tschannen M., Kramer T., Marti G., Heinzmann M., Wiatowski T. Heart Sound Classification Using Deep Structured Features. Proceedings of the Computing in Cardiology Conference (CinC).

[54] Gharehbaghi A., Lindén M. (2018). A deep machine learning method for classifying cyclic time series of biological signals using time-growing neural network. IEEE Trans. Neural Netw. Learn. Syst..

[55] Deperlioglu O., Kose U., Gupta D., Khanna A., Sangaiah A.K. (2020). Diagnosis of heart diseases by a secure Internet of Health Things system based on Autoencoder Deep Neural Network. Comput. Commun..

[56] LeCun Y., Bengio Y., Hinton G. (2015). Deep learning. Nature.

[57] Krizhevsky A., Sutskever I., Hinton G.E. ImageNet classification with deep convolutional neural networks. Proceedings of the Neural Information Processing Systems Foundation.

[58] Szegedy C., Ioffe S., Vanhoucke V. Inception-v4, inception-resnet and the impact of residual connections on learning. Proceedings of the AAAI.

[59] Russakovsky O., Deng J., Su H., Krause J., Satheesh S., Ma S., Huang Z., Karpathy A., Khosla A., Bernstein M. (2015). ImageNet Large Scale Visual Recognition Challenge. Int. J. Comput. Vis..

[60] Krishnan P.T., Balasubramanian P., Umapathy S. (2020). Automated heart sound classification system from unsegmented phonocardiogram (PCG) using deep neural network. Phys. Eng. Sci. Med..

[61] Schuster M., Paliwal K. (1997). Bidirectional recurrent neural networks. IEEE Trans. Signal Process..

[62] Neil D., Pfeiffer M., Liu S.-C. (2016). Phased LSTM: Accelerating Recurrent Network Training for Long or Event-based Sequences. Adv. Neural Inf. Process. Syst..

[63] Bentley G.N.P., Coimbra M., Mannor S. The Pascal Classifying Heart Sounds Challenge. http://www.peterjbentley.com/heartchallenge/index.html.

[64] Yaseen G.Y.S., Kwon S. (2018). Classification of heart sound signal using multiple features. Appl. Sci..

[65] Narváez P., Gutierrez S., Percybrooks W.S. (2020). Automatic Segmentation and Classification of Heart Sounds Using Modified Empirical Wavelet Transform and Power Features. Appl. Sci..

[66] Cho J., Lee K., Shin E., Choy G., Do S. (2016). How Much Data Is Needed to Train A Medical Image Deep Learning System to Achieve Necessary High Accuracy?. arXiv.

[67] Baydoun M., Safatly L., Ghaziri H., El Hajj A. (2020). Analysis of heart sound anomalies using ensemble learning. Biomed. Signal Process. Control.

[68] Thomae C., Dominik A. Using deep gated RNN with a convolutional front end for end-to-end classification of heart sound. Proceedings of the 2016 Computing in Cardiology Conference (CinC).

[69] Narváez P., Percybrooks W.S. (2020). Synthesis of Normal Heart Sounds Using Generative Adversarial Networks and Empirical Wavelet Transform. Appl. Sci..

[70] Ren Z., Cummins N., Pandit V., Han J., Qian K., Schuller B. Learning Image-based Representations for Heart Sound Classification. Proceedings of the 2018 International Conference on Digital Health.

[71] Humayun A.I., Khan T., Ghaffarzadegan S., Feng Z., Hasan T. (2018). An Ensemble of Transfer, Semi-supervised and Supervised Learning Methods for Pathological Heart Sound Classification. arXiv.

[72] Yosinski J., Clune J., Bengio Y., Lipson H. How transferable are features in deep neural networks?. Proceedings of the 27th International Conference on Neural Information Processing Systems.

